# Round the Hospitals

**Published:** 1920-11-20

**Authors:** 


### ROUND THE HOSPITALS.

The King held an Investiture in the Ball Room of Buckingham Palace on November 10, when His Majesty personally conferred decorations on members of the nursing services as follows: The Most Excellent Order of the British Empire, Military Division, Member—Miss Isabella Woodford, T.F.N.S., who also received the Royal Red Cross, First Class. Bar to the Royal Red Cross—Miss Marie Neville, Q.A.I.M.N.S.; Royal Red Cross and Bar—Miss May Hodgins, Q.A.I.M.N.S.; Royal Red Cross, First Class—Miss Georgina Jacob and Miss Fannie Manfield, Q.A.I.M.N.S.; Miss Marian Banister, Miss Winifred Blanchard, Mrs. Nora Dalrymple, Miss Isobel Drummond, Miss Mary Macdevitt, Q.A.I.M.N.S.R.; Miss Frances Wood, T.F.N.S.; Miss Flora Eadle, Mrs Alice Martin, Miss Alice Turnbull, Civil and War Hospital; Mrs. Laura Fisher, Australian A.N.S. Queen Alexandra received the Nursing Sisters together with Miss A. B. Smith, Matron-in-chief at Marlborough House, after the Investiture.

---

The Elizabeth Garrett Anderson Hospital is to be congratulated on the completion of the endowment of six more beds under the memorial scheme, viz., Oxford Colleges, St. Katharine's School, Hook Heath, Frances Mary Buss, Domestic Servants, Mary Scharlieb, and Civil Servants. The spirit in which these collections have been carried out is beyond praise, and the success which has attended the original scheme is an object-lesson in the art of applying to the right persons in the right way and at the right moment.

---

In the latest number of the College of Nursing *Bulletin* one of our constant readers, who does us the honour of finding The Hospital "excellent," gives voice to the gentle grumble, "the next week's issue always seems upon me before the last week's is properly read"! Now we have nothing to urge against a monthly issue of the *Bulletin*. We are quite certain that there is fully enough doing within the domain of the College of Nursing to call for a monthly *Bulletin* without poaching on our own wider domain of hospital and nursing interests. But we feel we may fairly point out to the Deal member of the College, who sighs for a monthly professional journal, that she has only to communicate with the publisher, and she can procure the monthly edition of The Hospital, handsomely got up in magazine form, at the price of one shilling. In this form it circulates in America and other places, and provides good reading to carry on through the month.

---

It is highly important that nursing sisters should realise as a body the significance of the League of Nations which meets at Geneva this week. The future of the world may be said to lie in their hands. From the purely professional standpoint the strength and unity of the League is the greatest force now existing in the sustained battle against disease for which the Red Cross Societies in every nation are girding themselves. It is an imperative duty on nurses in counsel to study individually and collectively the aims of the League of Nations, to follow its proceedings, and support it by their influence. The measure of strength which the League can attain to is the measure of the whole-hearted support it receives from groups of individuals in every nation which it represents. Few women exercise or ought to exercise more influence over the public than the nursing sisters called to alleviate mental and bodily suffering, and it will be a calamity if for lack of sound information on the subject they fail in their responsibilities towards the League. We wish the aims of the League could be powerfully expounded in every centre of the College of Nursing.

---

Queen Mary's London Needle Guild had a good display at the Imperial Institute early this week. It is an admirably organised society, and the spirit which pervades its supporters can be known by its fruits. The group presided over by the Queen in person sent in nearly 18,000 articles, among which the white coverlets, delicately crocheted in fine wool and lined with pale blue and pale pink satin, attracted much attention as Her Majesty's own handiwork. They are to be used by nursing associations in the East and West of London respectively. Many members of the Royal Family have been pressed into service for the Guild. The King sends large bundles of good flannel shirts, and the Prince of Wales too, while there are piles of warm vests and socks from the other Princes. Princess Mary, who presides over a group of her own, has provided a fine array of boots and many complete outfits for lying-in mothers, as well as for girls going to their first situation. In distributing the gifts special regard is to be had to the needs of ladies with small fixed incomes who have suffered so much in the rise of prices, and we trust the managers of the Nation's Tribute Fund for Nurses may be privileged to turn some of these handsome gifts in the direction of pensioned nursing sisters.

---

Another nurses' home is to be founded as a War Memorial, and will be among the most famous of those already planned. It is to be built in connection with the memorial to the 63rd Royal Naval Division. An appeal has been circulated by Brigadier-General Asquith for £8,000 for a memorial to be erected in the Mall at the foot of the Duke of York's steps, and the balance of the money subscribed is to be devoted to a nurses' home. The fund will be kept open till January 15, and subscriptions, however small, may be sent to the R.N.O. Memorial Fund, Cornwall House, Stamford Street, S.E.

---

Mr. Fred Smith, of the Highfield, Uffculme, and Sorrento Hospitals, Moseley, Birmingham, in referring to a paragraph in this column in reference to Mrs. E. Lloyd, who has been recently appointed as

**ROUND THE HOSPITALS**—(*continued*).

Food Supervisor at the General Hospital, Birmingham, in which it was stated that Highbury Hospital is a "V.A.D." hospital, reminds us that the Hospital is now the Highbury Orthopædic Hospital, and has been under the control of the Birmingham War Pensions Committee for the Ministry of Pensions since March 1919. Previous to that date and during the period of the war, the hospital was a V.A.D. hospital.

We have received from the Scientific Press, Ltd., a volume of interesting "Lectures to Nurses in Training" (2s. 6d. net), by Miss M. Vivian, A.R.R.C., late Matron of Victoria Hospital, Deal, and Princess Christian's Hospital, Weymouth. They have a double purpose, first to help the candidate to prepare in the best way for entering hospital and make a good choice of a training-school, next to help the young nurse over the first two years of her training. There is wisely no attempt to give instruction about the manner in which the practical duties of nursing are performed. The single aim of setting nursing ethics before the probationer has been kept in view. We know of no book which exactly fills the place of this pleasantly written and sound volume. Many matrons of smallish hospitals will find it invaluable as a groundwork for addresses to their own probationers. The spirit in which it is conceived may be judged from the following remark taken almost haphazard: "Every probationer takes her part in creating the atmosphere of a ward, and one unsympathetic, selfish nurse in a ward will so help to lower its tone that the patients become discontented and unhappy, themselves hardly knowing the reason."

Wakefield has decided on a new nurses' home as the city war memorial, and the nursing staff of the Clayton Hospital lately held a very successful sale of work and American tea, with various attractive side-shows, in aid of the furnishing fund. Lady Kathleen Pilkington opened the proceedings, which were very well attended, and the sum of £346 was handed over to the War Memorial Committee to purchase furniture for the nurses' home.

Very necessary reforms in the nurses' hours and way of living at the Croydon General Hospital have long been delayed owing to lack of accommodation. The Secretary is appealing for funds to provide the much-needed extra bedrooms for the staff, as well as to open two small additional wards. The British Red Cross Society has promised a grant of £1,000 towards the extension, and the work is already in hand, so that there is some hope the nurses will enter into comfortable quarters early next year. At least another thousand must be collected to earn the Red Cross grant.

The resignation of one of the nurses at the Pontypridd Cottage Hospital has led to the appointment of a committee of five to revise the salaries of the whole staff. Mr. Cook, who proposed this, made the exquisite remark that "the sisters and nurses must be treated as human beings as well as the patients whom they attended so well."

An important conference has been held in the Council Chamber of the Gosport District Council to consider an offer from the Hants County Nursing Association of the sum of £3,000 towards the establishment in Gosport of a maternity training home for midwives to work in the county. The Rev. Guy Landon, Rector of Gosport, presided, and Mrs. Rawstorne represented the County Nursing Association. The general opinion of the meeting was very favourable to the scheme, and it was resolved to lay it before the War Memorial Hospitals Committee for further consideration and report. If the new maternity home could be incorporated as part of the War Memorial it would undoubtedly secure it widespread support, and there seems hope that this plan may be adopted. In the event of this falling through, the Hants County Nursing Association will probably combine with the Victoria Nursing Society for the training of midwifery nurses, and in that case would allocate the £3,000 to provide a good nurses' home for those in training. Midwives are so much needed in the county that we trust financial difficulties may be speedily overcome and the work commenced.

According to the latest report of the Durham County Midwives' Inspector, the "handy woman" in certain districts continues her neighbourly attentions in spite of the fact that a trained woman is now available. Often she gets a much higher fee than is charged by the qualified woman for nursing the case during the puerperium. Prejudice dies hard; but the inspector thinks the mother is realising that the honourable position of grandmother does not necessarily bring knowledge, and that a woman (even though she is young and single) who has been specially prepared for the work is more competent than the best of well-meaning neighbours. Some mothers have observed that they would not like to go back to the old order of things after having once been attended by a trained woman.

At the Maternity and Child Welfare Committee of the Barnes Urban District Council a letter was read from the Surrey County Council respecting the appointment of Miss Stanley as health visitor. The County Council considered the certificate of the C.M.B. an essential qualification for the appointment, and as Miss Stanley has not this qualification the County Council was unable to approve her appointment. The Maternity Committee have, however, informed the County Council that it is extremely difficult to obtain health visitors who are qualified midwives; they pointed out that another health visitor has the required qualification and that both health visitors are doing their work in a very satisfactory manner. It is evident that health visiting is in a transition state, and that there are more good openings for thoroughly qualified women than there are candidates.

